# Prospective cohort study on non-specific symptoms, cognitive, behavioral, sleep and mental health in relation to electronic media use and transportation noise among adolescents (HERMES): study protocol

**DOI:** 10.12688/openreseurope.17667.1

**Published:** 2024-06-19

**Authors:** Hamed Jalilian, Nekane Sandoval-Diez, Valentin Jaki Waibl, Michael Schmutz, Simona Trefalt, Nasrullah Arslan, Adriana Fernandes Veludo, Laura Tincknell, Irina Wipf, Lena Steck, Stefan Dongus, Agnieszka Jankowska, Gabriela P. Peralta, Kinga Polanska, Maja Popovic, Milena Maule, Patricia de Llobet, Monica Guxens, Martin Röösli

**Affiliations:** 1 Department of Epidemiology and Public Health, Swiss Tropical and Public Health Institute, Allschwil, 4123, Switzerland; 2 University of Basel, Basel, 4003, Switzerland; 3 Meteotest AG, Bern, 3012, Switzerland; 4 Department of Environmental and Occupational Health Hazards, Nofer Institute of Occupational Medicine, Lodz, 91-348, Poland; 5 Instituto de Salud Global Barcelona, Barcelona, Spain; 6 Universitat Pompeu Fabra, Barcelona, Spain; 7 Spanish Consortium for Research on Epidemiology and Public Health (CIBERESP), Instituto de Salud Carlos III, Madrid, Spain; 8 Unit of Cancer Epidemiology, Department of Medical Sciences, University of Turin and CPO Piemonte, Turin, Italy; 9 Department of Child and Adolescent Psychiatry/Psychology, Erasmus MC, University Medical Centre, Rotterdam, The Netherlands

**Keywords:** Electronic media, electromagnetic field, transportation noise, sleep, mental health, cognition

## Abstract

Electronic media (eMedia) devices along with exposure to transportation noise are integral to the daily routines of adolescents. The concerns associated with excessive eMedia usage extend beyond sleep deprivation to include the heightened exposure to radiofrequency electromagnetic fields (RF-EMF) emitted by these wireless devices. The aim of HERMES (Health Effects Related to Mobile PhonE Use in AdolescentS) study is to better understand biophysical and psychological pathways in relation to eMedia, RF-EMF exposure use and transportation noise that may effect on cognitive, behavioral, sleep and mental health, as well as non-specific symptoms.

Following two previous HERMES cohorts conducted between 2012 and 2015 we have initiated the third wave of HERMES study as a prospective cohort with intermediate (every four months) and one year follows-up. Eligible participants are adolescents attending 7 ^th^ or 8 ^th^ school grades in Northwest and Central Switzerland. Baseline examinations are a questionnaire on eMedia usage and selected health outcomes, as well as computerized cognitive tests. In addition, parents/guardians are asked to fill in a questionnaire about their child’s health and potential eMedia use determinants. Far-field RF-EMF exposure and transportation noise at the place of residence and school are predicted based on a propagation model. Cumulative RF-EMF brain dose is calculated based on self-reported eMedia use, mobile phone operator data, and RF-EMF modelling. A follow-up visit is conducted one year later, and two interim questionnaires are sent to adolescents to be completed at home. Between baseline and 1-year follow-up, a subsample of 150 study participants is invited to collect personal RF-EMF measurements as well as sleep and physical activity data using accelerometers.

This new recruitment wave of HERMES study provides a greater understanding of causal pathways between eMedia, RF EMF, and transportation noise exposure and their effects on health outcomes, with relevant implications for both governmental health policy and lay people alike.

## Plain Language Summary

Use of electronic media (eMedia) devices such as mobile phones as well exposure to transportation noise are inseparable parts of adolescents’ everyday life. The excessive use of eMedia can be of concern when it results in sleep deprivation, but also when it leads to an increase in exposure to radiofrequency electromagnetic fields (RF-EMF) emitted by wireless devices. These exposures could potentially affect cognition, and mental health resulting in behavioral problems of adolescents.

This study is part of a large research effort within the project GOLIAT aiming to understand the relationship of eMedia use, RF EMF exposure, and transportation noise exposure with cognitive, behavioral, sleep and mental health, as well as non-specific symptoms of Swiss adolescents. In this research, we recruit adolescents in secondary school and use questionnaires, cognitive tests, measurement devices, and mobile applications to collect information regarding exposure and outcomes at two time points, at baseline and one year later.

## Introduction

Electronic media (eMedia) form an intrinsic component in the everyday life of Generation Z (born after 1997), often referred to as iGen for their extensive use of communication devices ( Chassiakos & Stager, 2020). The term eMedia refers to communication occurring over the internet or mobile networks using mobile phones, computers, tablets, wearables, or other digital devices ( Ekhareafo, 2023). Intensive eMedia usage has been linked to various non-specific health outcomes such as fatigue and headache as well as mental, cognitive and sleep health issues ( Bodewein *et al.*, 2022; Carter *et al.*, 2016; Girela-Serrano *et al.*, 2022; Ishihara *et al.*, 2020).

Causal pathways of the relationships between eMedia usage and mental health are unclear and evidence is inconclusive. However, a recent systematic review found suggestive but limited evidence on the association between poorer mental health among children and adolescents with greater use of mobile phones/wireless devices ( Girela-Serrano *et al.*, 2022). Broadly, research into health effects of eMedia and mobile phone usage can be viewed through two important distinct pathways: *biophysical* effects related to radiofrequency electromagnetic fields (RF-EMF) emitted by devices, and *psychological (non-biophysical)* aspects related to potential effects of device usage not linked to RF-EMF (e.g., addiction, sleep deprivation) ( Eeftens *et al.*, 2023a; Lissak, 2018a; Singh & Kapoor, 2014; Thomée, 2018).

The biophysical pathway postulates that RF-EMF radiation emitted by digital devices interacts with the body and brain and causes non-specific health symptoms (such as headache and lack of concentration), affects cognition, and may be detrimental to mental health ( Baliatsas *et al.*, 2012; Kim *et al.*, 2019; Röösli *et al.*, 2024). These concerns are significantly more remarkable among children because of the potentially greater susceptibility of their developing nervous systems, higher brain tissue conductivity for RF-EMF, and longer lifetime exposure compared to adults ( Kheifets *et al.*, 2005; Kim *et al.*, 2019). A recent review of epidemiological and experimental studies on the effects of RF-EMF on children and adolescents found that the body of evidence for any effects was inconclusive and of low quality ( Bodewein *et al.*, 2022).

The RF-EMF exposure is heavily user-dependent and therefore makes exposure assessments a particular challenge for studies examining the relationship of RF-EMF exposure and health outcomes. Recent studies suggest that sources close to the body emitting RF-EMF (uplink) such as mobile phones contribute approximately 70% to the whole body RF-EMF dose and 85% to the whole brain dose ( van Wel *et al.*, 2021). Two recent studies observed that native phone calls contribute to the majority of the total daily RF-EMF brain dose ( Birks *et al.*, 2021; Eeftens *et al.*, 2023b). Thus, exposure may vary considerably depending on the type of device and usage.

The psychological pathways provide multiple non-RF-EMF hypotheses to possible correlations between eMedia use and its effects on cognition and mental health. The dopamine metabolism of children is more sensitive than that adults, which leads to stronger activations of the reward pathways and therefore result in higher risk of behavioral addiction to eMedia ( Lissak, 2018b). The effects of eMedia usage on sleep postulate additional consequences. Use of eMedia in the evening may postpone falling asleep or maybe waking up by calls and messages during the night. This pattern can eventually result in sleep deprivation and insomnia, which is suggested to act as a mediator on mental health outcomes such as depression, anxiety, as well as behavioral problems and cognition ( Carter *et al.*, 2016; Girela-Serrano *et al.*, 2022; Lund *et al.*, 2021).

Although it is assumed that reducing environmental noise below the harmful thresholds (from 40 dB night time aircraft noise to 54 dB daytime railway noise) recommended by the World Health Organization (WHO) will prevent any potential negative effects ( WHO, 2018), recent scientific evidence demonstrates that even noise levels below these limits may also have a negative impact on health and well-being ( Sørensen *et al.*, 2024). Children and adolescents are considered to be among the most vulnerable groups to negative health impacts from noise ( Eulalia, 2020) because they are in an essential period of learning and development and may lack coping mechanisms over background noise levels. Long-term memory reading, language skills, and executive functioning have been negatively associated with noise exposure in children ( Clark & Paunovic, 2018; Raess *et al.*, 2022; Thompson *et al.*, 2022). A lower self-reported sleep quality ( Basner & McGuire, 2018; Pérez-Crespo *et al.*, 2023) and hyperactivity and attention problems ( Schubert *et al.*, 2019) have also been linked to noise exposure among children and adolescents. However, the majority of this evidence stems from highly specific research focusing only on aircraft noise exposure, undertaken in a specific demographic settings, or applying mostly cross-sectional designs ( Clark & Paunovic, 2018). Therefore, more research is needed addressing other long-term effects, conducted across broader child age ranges (e.g. in adolescents), employing more robust methodological designs, and incorporating both home and school exposures.

The HERMES1 and HERMES2 studies (Health Effects Related to Mobile phone usE in adolescentS), conducted between 2012 and 2015, added a large body of evidence to the literature on RF-EMF, noise exposures, and health outcomes. These cohorts found that at that time mobile phone use was the most relevant contributor to cumulative RF-EMF dose ( Roser *et al.*, 2015a; Roser *et al.*, 2017). Cognitive functions were studied in HERMES cohorts and Foerster *et al.* (2018) reported that exposure to RF-EMF might affect brain processes such as cognitive functions that involve brain regions mostly exposed during mobile phone use ( Foerster *et al.*, 2018). In cross-sectional analyses, Roser *et al.* (2016b) found behavioral problems to be associated with several self-reported wireless device use measures but not with operator-recorded mobile phone use, while concentration capacity was associated with both several self-reported and operator-recorded exposures. However, due to the lack of associations in longitudinal analyses after a one year of follow-up, this study considered information bias and reverse causality as most likely explanations for the observed results ( Roser *et al.*, 2016b). Additional research in the HERMES cohorts showed an association between nighttime use of mobile phone, but not RF-EMF exposure, and increased health symptoms such as tiredness, rapid exhaustibility, headache and physical ill-being ( Roser *et al.*, 2016a; Schoeni *et al.*, 2015; Schoeni *et al.*, 2016; Schoeni *et al.*, 2017).

Given the rapid changes in eMedia usage patterns since the previous HERMES cohorts and the lack of longitudinal research, HERMES3 plans to conduct the third wave cohort, which aims at collecting data that more accurately reflects the current state of eMedia and mobile phone use in adolescents. The HERMES3 cohort is part of GOLIAT project (5G Exposure, Causal Effects, and Risk Perception through Citizen Engagement), a five-year European project that aims to monitor RF-EMF exposure, particularly from 5G, provide novel insights into its potential causal health effects, and understand how exposures and risks are perceived and best communicated using citizen engagement ( https://www.isglobal.org/en/-/5g-exposure-causal-effects-and-risk-perception-through-citizen-engagement). Within the GOLIAT project, HERMES3 together with six other cohorts from Spain, Italy, Poland, the Netherlands, Japan and South Korea is expected to considerably increase the knowledge on the effects of eMedia use by differentiating between biophysical and psychological (non-biophysical) pathways.

Specific aims of the HERMES study are

- To explore RF-EMF exposure of adolescents and calculate cumulative source specific RF-EMF dose based on personal measurements, eMedia usage and modeling of fixed site transmitter emissions.
- To explore whether cumulative RF-EMF exposure is associated with cognitive, behavioral, sleep and mental health, as well as non-specific symptoms in adolescents (biophysical pathway).
- To explore what aspects and patterns of eMedia use, such as nighttime usage or what type of social networking sites (SNS) use are associated with cognitive, behavioral, sleep and mental health, as well as non-specific symptoms in adolescents (psychological pathway).
- To explore to what extent the biophysical (RF-EMF) and the psychological pathways interact with each other in any observed associations between eMedia use and adolescent outcomes.
- To explore whether transportation noise exposure is associated with cognitive, behavioral, sleep and mental health, as well as non-specific symptoms in adolescents (biophysical pathway).

## Protocol

### Design

**Figure 1.**
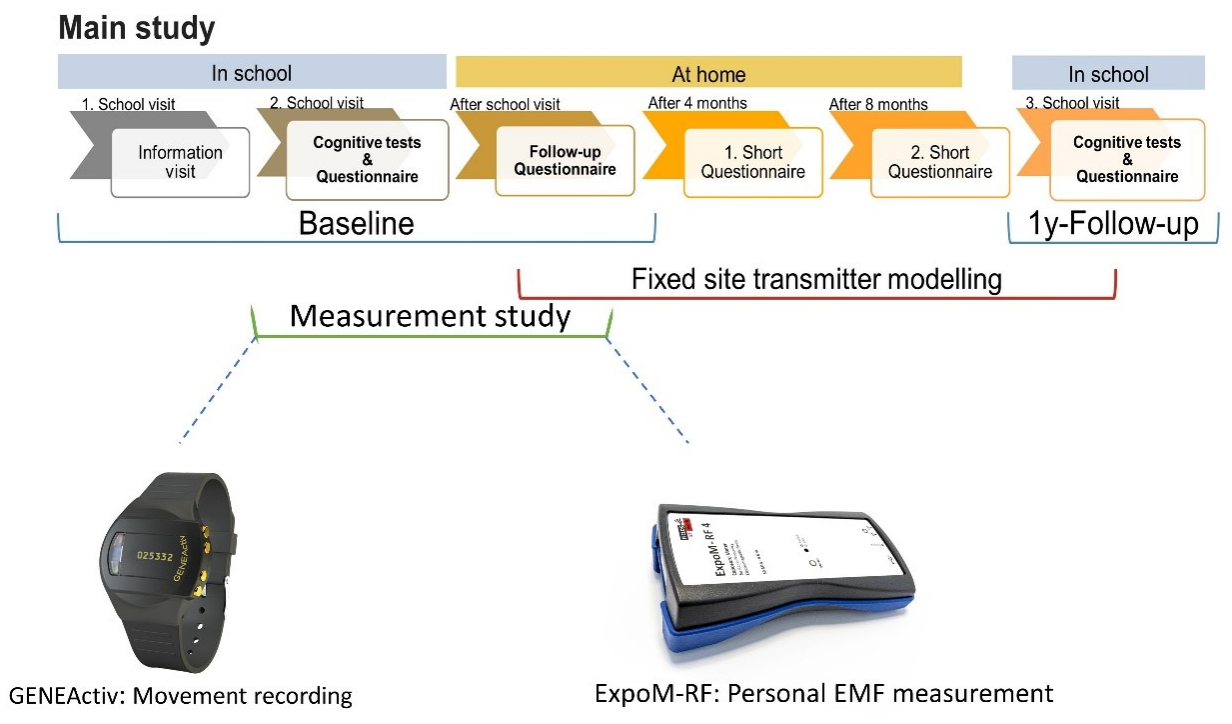
HERMES3 study design: the measurement study takes place in a subsample of 150 adolescents.

HERMES3 is a prospective cohort study with one-year follow-up period and a nested measurement study in a subsample of study participants ( Figure 1). Additionally, in HERMES3 we consider two intermediate follow-ups, here after called interim assessments, every 4 months to support the cohort.

### Population, inclusion and exclusion criteria

This cohort aims to enroll approximately 900 adolescents, out of which 150 concurrently take part in the nested measurement study, in Central and Northwest Switzerland (i.e. in the canton of Aargau, Solothurn, Basel-City and Basel-Country). Eligible participants are from 7 ^th^ and 8 ^th^ grade classes willing to participate and aged between 11–14 years old at the time of recruitment (2023–2024). Additional inclusion criteria for students include the ability to speak German (assessed by the teacher) or English (in international schools), and the ability to give consent. Furthermore, access to school computers is necessary for the completion of the questionnaire and cognitive testing.

### Recruitment, screening and informed consent procedure

We included all the public schools and private schools that are located in Central and Northwestern part of Switzerland. School directors are contacted directly to recruit adolescents. If school directors and 7 ^th^ or 8 ^th^ grade teachers agree to participate, a first school visit is planned in which trained researchers introduce the study aims to the students and the teachers ( Figure 1). During this visit, additional study details and informed consent forms are distributed among the adolescents. Both adolescents and their primary guardians should provide their written consent in order to be eligible to participate in the study. There is no gender restriction for participating in the main study or measurement studies and anyone who meets the inclusion criteria could participate in the main and nested study.

The consent form also requests contact information of the participant and the primary guardian willing to participate in the measurement study. Guardians have the option to receive a paper questionnaire instead of an electronic questionnaire. Additionally, a separate form is distributed to inquire whether study participants and their guardians are willing to provide access to their mobile phone records from the provider company.

In the following four to six weeks after the initial school visit ( Figure 1), the baseline assessment takes place during the second school visit. To incentivize adolescents to take part in the main study, all participants receive a 5 CHF voucher for a convenience shop.

### Assessment tools

***Exposure assessment***

**eMedia usage**

1.   eMedia Questionnaire: A survey on self-reported mobile phone screen time as well as eMedia use, which includes the assessment of differentiated usage by various electronic devices including smart phone, cordless phones, laptops and computers, and wearable devices.

2.   Operator recorded data: Mobile phone use that includes data on networks technology, data traffic and calls duration are collected from mobile phone operators.

3.   SleepMedia log: eMedia usage also is examined using the self-reported SleepMedia log among nested measurement study participants to collect more details on eMedia activities.

**RF-EMF exposure**

1.   NISMap: Far field exposure from mobile phone base stations is estimated using a spatiotemporal model of RF-EMF exposure, called NISMap ( Bürgi *et al.*, 2010). Briefly, the model is based on accurate operation parameters of all stationary transmitters of mobile communication base stations, radio broadcast and television transmitters. RF-EMF exposure at the address of residence and school is then estimated using established propagation algorithms.

2.   ExpoM-RF4: Personal RF-EMF measurements are collected among the nested measurement study participants using a portable measurement device (ExpoM-RF4, Fields at work GmbH, Switzerland) ( Fields at Work, 2023). The ExpoM-RF4 device measures several RF-EMF bands with high accuracy and sensitivity, allowing a detailed characterization of exposure from the major broadcasting and wireless communication services ( Table 1).

**Table 1.** Bands descriptions, center frequencies, bandwidths and category (band summation) of ExpoM-RF 4 measurement device *.

N°	Description	Center frequency (MHz)	Bandwidth (MHz)	Category
**1**	FM Radio	97.75	35	broadcast
**2**	DAB/DAB+	202	75	broadcast
**3**	Polycom / TETRAPOL	385	35	broadcast
**4**	TETRAPOL, amateur, ISM 433	422.5	35	broadcast
**5**	PMR/PAMR (Betriebsfunk)	452.5	35	broadcast
**6**	DVB-T (1)	507.5	75	broadcast
**7**	DVB-T (2)	583.5	75	broadcast
**8**	DVB-T (3)	659.5	75	broadcast
**9**	Mobile 700 uplink	718	35	uplink
**10**	Mobile 700 TDD	748	35	time division duplex (TDD)
**11**	Mobile 700 downlink	770.5	35	downlink
**12**	Mobile 800 downlink	808.5	35	downlink
**13**	Mobile 800 uplink	847	35	uplink
**14**	Mobile 900 uplink	897.5	35	uplink
**15**	Mobile 900 downlink	942.5	35	downlink
**16**	Mobile 1400 supplementary downlink	1479.5	75	downlink
**17**	Mobile 1800 uplink	1747.5	75	uplink
**18**	Mobile 1800 downlink	1842.5	75	downlink
**19**	DECT	1897.5	35	Cordless phone
**20**	Mobile 2100 uplink	1957	75	uplink
**21**	Mobile 2100 downlink	2145	75	downlink
**22**	ISM 2.4 GHz	2438	100	Wi-Fi
**23**	Mobile 2600 uplink	2535	75	uplink
**24**	Mobile 2600 TDD	2592.5	35	TDD
**25**	Mobile 2600 downlink	2657	75	downlink
**26**	Mobile 3500 (1)	3475	100	TDD
**27**	Mobile 3500 (2)	3605	100	TDD
**28**	Mobile 3500 (3)	3735	100	TDD
**29**	Wi-Fi 5 GHz (1)	5200	100	Wi-Fi
**30**	Wi-Fi 5 GHz (2)	5325	100	Wi-Fi
**31**	Wi-Fi 5 GHz (3)	5450	100	Wi-Fi
**32**	Wi-Fi 5 GHz (4)	5575	100	Wi-Fi
**33**	Wi-Fi 5 GHz (5)	5700	100	Wi-Fi
**34**	Wi-Fi / SRD 5.8 GHz (1)	5825	100	Wi-Fi
**35**	Wi-Fi / SRD 5.8 GHz (2)	5950	100	Wi-Fi

* Frequency range: 80–6000 MHz; detection limit: 0.0019

3.   Brain RF-EMF dose: Cumulative individual RF-EMF brain dose is derived by combining the participant questionnaire data on exposure relevant activities with RF-EMF environmental exposure and dosimetric simulations that are part of the EU project GOLIAT following an updated approach as described in ( van Wel *et al.*, 2021) and applied in ( Birks *et al.*, 2021) or ( Eeftens *et al.*, 2023b). RF-EMF brain dose model considers RF-EMF exposure relevant behaviors and exposure circumstances from near-, intermediate- and far-field sources. Near field refers to the use of RF-EMF–emitting devices close to the body (e.g., mobile phones), far field refers to the environmental RF-EMF exposure (e.g., from fixed-site transmitters, people using mobile phones nearby), and intermediate field refers to personal exposure to Wi-Fi router signals ( Foerster *et al.*, 2018).

**Noise exposure**

1.   sonBASE 2015 model: Exposure to road, railway, and aircraft noise is determined at each participants’ residence and school location using the updated sonBASE 2015 model ( BAFU, 2018). This three-dimensional source-propagation model considers the geometry of the noise emission sources and novel road traffic emission modelling with ten separate vehicle categories, detailed traffic count data, and the 3D building dataset from the Federal office of Topography (Swisstopo) with more accurate apartment locations within buildings.

2.   Sound level meter: To describe accurately and analyze the contribution of transportation noise exposure at school we used a Noise Sentry RT type-II sound level meter data logger (Convergence Instruments, Sherbrooke, QC, Canada) ( Bruno, 2014).

***Outcomes assessment***

**Cognitive function**

Executive functioning is measured using a computerized, standardized cognitive test battery (Creyos, formerly Cambridge Brain Sciences) ( Eeftens *et al.*, 2023a). Tests cover different brain regions and hemispheres associated with various aspects of cognition, including memory, reasoning, verbal ability, and attention. A description of the selected six tests is shown in Table 2.

**Table 2.** Description and screenshots of the cognitive test battery.

**1**	**Spatial span test (based on Corsi block tapping test)** **Outcome**: Spatial short term memory **Duration**: ~90 seconds (self-adaptive, until 3 mistakes are made) **Brain area**: *Right* mid-ventrolateral area, parieto-occipital regions **Description**: The cognitive system that allows for temporary storage of spatial information in memory. Spatial short-term memory deals with the relationships between objects in space, as opposed to remembering the specific order of numbers or words involved in verbal short-term memory. **Score**: The maximum length of a sequence that is correctly repeated	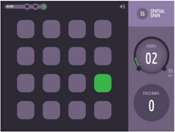
**2**	**Odd One Out Task** **Outcome**: Deductive reasoning **Duration**: 180 seconds **Brain area:** Anterior frontal cortex, anterior cingulate, anterior insula / frontal operculum, inferior frontal sulcus, pre-supplementary motor area, intraparietal sulcus **Description:** The core cognitive ability to apply rules to information in order to arrive at a logical conclusion. **Score:** The number of correct answers – the number of wrong answers	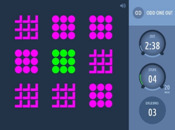
**3**	**Grammatical reasoning test** **Outcome:** Verbal reasoning **Duration:** 90 seconds **Brain area:** frontal operculum, posterior temporal lobe, superior parietal lobe, dorsal prefrontal cortex, ventral prefrontal cortex **Description:** The ability to quickly understand and make valid conclusions about concepts expressed in words. **Score:** The number of correct answers – the number of wrong answers	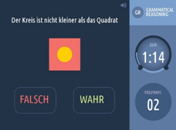
**4**	**Digit span task** **Outcome**: Verbal, short term memory **Duration**: ~90 seconds (self-adaptive, until 3 mistakes are made) **Brain area:** Mid-ventrolateral prefrontal cortex, *left* temporo-parietal lobe, basal ganglia **Description:** Short-term memory is the cognitive system that allows for temporary storage of information in memory. Verbal short-term memory deals with numbers or words in a specific order, as opposed to spatial short-term memory. **Score:** The maximum length of a sequence that is correctly repeated	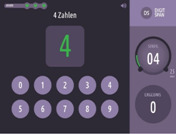
**5**	**Double trouble task (based on Stroop Colour-Word Test)** **Outcome**: response inhibition **Duration**: 90 seconds **Brain area:** *Right* prefrontal cortex. dorsolateral frontal cortex, *left* inferior frontal gyrus, dorsal striatum **Description:** The ability to concentrate on relevant information in order to make a correct response despite interference or distracting information. **Score:** The number of correct answers – the number of wrong answers	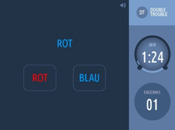
**6**	**SART (Sustained Attention to Response)** **Outcome**: sustained attention **Duration**: 4 minutes **Brain area:** frontal and parietal cortical areas, mostly in the *right* hemisphere. **Description:** The ability to maintain concentration for the whole 4 minutes so that one does not click/tap/press after a number 3. **Score:** The number of correct answers – the number of wrong answers	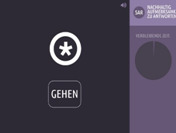

**Behavioral problems**

The Strengths and Difficulties Questionnaire (SDQ): A validated questionnaire used to assess general behavioral difficulties in adolescents ( Birks *et al.*, 2017; Divan *et al.*, 2012; Goodman, 1997; Roser *et al.*, 2016b; Toledano *et al.*, 2018). It consists of 25 items scored on a 3-point Likert scale, and can be scored either with five-factor subscales (emotional difficulties, conduct difficulties, hyperactivity difficulties, peer problem difficulties, and prosocial behavior) or broadly in two subscales (internalizing and externalizing symptoms).

**Mental health**

1.   The Generalized Anxiety Disorder-7 Questionnaire (GAD-7): This scale is a self-reported questionnaire used to assess the severity of generalized anxiety disorder. It consists of seven items, each corresponding to one of the diagnostic criteria for generalized anxiety disorder. Respondents rate each item on a scale from 0 to 3 based on how frequently they have experienced the symptom over the past two weeks (0 = Not at all, 1 = Several days, 2 = More than half the days, 3 = Nearly every day). The total score ranges from 0 to 21, with higher scores indicating more severe anxiety symptoms ( Toledano *et al.*, 2018).

2.   Patient Health Questionnaire-9 (PHQ-9): A concise and widely used screening tool designed to assess the severity of depressive symptoms in individuals over the past two weeks using nine items. Respondents rate the frequency of each symptom on a scale from 0 to 3. Scores range from 0 to 27, with higher scores indicating more severe depression ( Kroenke *et al.*, 2001).

3.   Eating Disorder Examination Questionnaire-7 (EDE-Q7): This is a validated short-form screening tool used to assess body dissatisfaction and has been used with adolescents. It consists of seven items that cover aspects such as restraint, eating concern, weight concern, and shape concern. Respondents rate the frequency of their experiences on a scale from 0 to 6 over the past 28 days. Higher scores indicate more severe eating disorder symptoms ( Grilo *et al.*, 2015; Machado *et al.*, 2020).

4.   Non-Suicidal Self Injury: Due to the lack of short-form validated measures of Non-Suicidal Self Injury (NSSI), this study uses an adaptation of the single-item question from the Child & Adolescent Self-Harm in Europe study ( Madge *et al.*, 2008).

**Non-specific symptoms**

1.   Headache Impact Test-6 (HIT-6): We ask about headache using HIT-6, which is a validated and broadly used questionnaire ( Piebes *et al.*, 2011).

2.   von Zerssen Complaints checklist: Selected items of this tool are used to assess non-specific somatic symptoms including tiredness, sense of balance, exhaustibility, lack of energy, and lack of concentration ( Von Zerssen & Petermann, 2011).

**Sleep**

1.   SleepMedia log: In addition to eMedia usage, SleepMedia log is collecting sleep data that includes items regarding subjective sleep disturbances (e.g. waking up due to noise), subjective sleep duration (time between sleep onset and waking up), subjective sleep onset and wake up time, subjective sleep onset latency (time between lying down in bed and falling asleep, in minutes) and subjective daytime sleepiness.

2.   Sleep Disturbance Scale for Children (SDSC): Sleep disturbance is assessed using an adapted version of SDSC ( Bruni *et al.*, 1996; Guxens *et al.*, 2012). This questionnaire consisting of 26 Likert-type items grouped into six subscales that designed both to evaluate specific sleep disorders in young children, and to provide an overall measure of sleep disturbance suitable for use in clinical screening and research.

3.   Objective sleep measures: Additionally, for those participating in the nested study, sleep and physical activity are physiologically monitored according to an accelerometer data (GENEActiv, Activinsights, UK) ( Activinsights, 2023). The GENEActiv device monitor movement in everyday living behaviors, and provide unfiltered data as well as algorithms for digital clinical measures comparable to previous research ( Germini *et al.*, 2022). Participants wear this device continuously for a period of time and data is downloaded using the GENEActiv software application. For each day, calculated objective data includes total sleep time (time between falling asleep and final awakening from which the time spent awake in between is subtracted, in hours), sleep efficiency (total sleep time divided by total time in bed, in %), sleep onset latency (time between lying down in bed and falling asleep, in minutes), and wake after sleep onset (time awake between falling asleep and final awakening, in minutes).

**Health related quality of life**

The KIDSCREEN-10 Index: This is a questionnaire used to assess health-related quality of life (HRQoL) in children and adolescents. It consists of 10 items covering various aspects of well-being, including physical, emotional, social, and school functioning. Respondents rate each item on a 5-point Likert scale, with higher scores indicating better HRQoL ( Mireku *et al.*, 2019).

***Baseline and co-variables***

- 1.   HERMES3 collects the baseline factors that have potential to be a confounder or relevant eMedia use determinants at baseline and one-year follow-up from the participating adolescents and guardians ( Table 3).

**Table 3.** Overview of the sources for various domains of information.

Theme	Items	Respondent
**Sociodemographic**	Age	Adolescent
	Sex	Adolescent
	Family structure	Adolescent
	Socioeconomic status	Guardian
	Migratory status	Guardian
**Behavioral**	Alcohol use	Adolescent
	Cigarette use	Adolescent
	e-cigarette use	Adolescent
	Marijuana use	Adolescent
	Physical activity	Adolescent
**Medical illness**	Diagnosed physical illness or disability	Guardian
	Diagnosed mental illness or disability	Guardian
	Medication use	Guardian
**Noise**	Noise annoyance, noise sensitivity, bedroom orientation to street	Adolescent
**Housing ** **characteristics**	Factors relevant for RF-EMF modelling	Guardian
	Factors relevant for noise modelling	Guardian
	Factors related to sociodemographic factors including address	Adolescent/guardian
**Other ** **environmental ** **exposures**	Spatial modelling of green space, based on normalized difference vegetation index (NDVI) and on land use mapping ( Vienneau *et al.*, 2017), plus long-term air pollution levels (NO2, PM2.5) ( De Hoogh *et al.*, 2019).	Modelling based on geocode

- 2.   Primary guardians questionnaire: The primary guardians of the study participants are asked to fill in a questionnaire on eMedia use as well as RF-EMF, and noise exposure sources at home, and relevant covariates related to their child’s health. The factors are age, sex, nationality, school level, environmental exposures, physical activity, alcohol consumption and educational level of the parents ( Table 3).

***Interim questionnaire***

This questionnaire includes items on eMedia usage, social media and content, screen time, reading behavior, sleeping behavior and mental health.

***Diary app***

**Figure 2.**
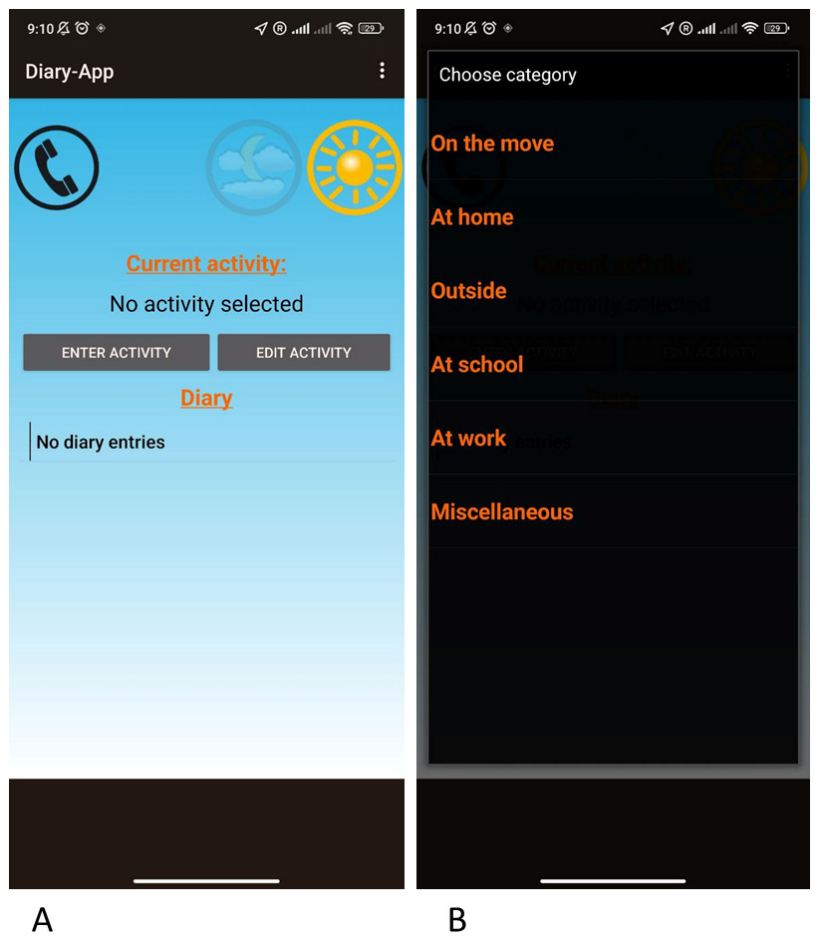
Two screen shots of diary app indicating the ( **a**) first page and ( **b**) activity page.

A smartphone equipped with a diary app enables participants to record time spent at home, school, public transport, outdoors, and miscellaneous ( Figure 2). It is kept on flight mode and is only used for diary data collection.

### Study procedures

***Main study***

During the second school visit ( Figure 1), participants take the cognitive tests on school computers during school hours and they are instructed and supervised by members of the study team. Additionally, they are asked to complete the first part of the online questionnaires ( Table 4).

**Table 4.** School and home questionnaires content.

Domain	Content
***School***	
**Personal information**	These questions cover personal characteristics of participants
**Public transport**	To assess the amount of time each participant spends in a given public mode of transport
**RF-EMF exposure-mobile phone**	To assess usage/handling habits (for example 5G compatibility of phone, voice calls, internet activities, social media, etc.). as well as phone accessory usage (for example headphones)
**RF-EMF exposure-Laptop/Tablet**	To assess personal usage time (i.e. how long, when, where) and activities. Additionally, wireless headphone usage asked
**RF-EMF exposure-cordless phone**	To indicate cordless phone pattern usage
**RF-EMF-other sources close to body**	To indicate usage pattern of electronic accessories such as smartwatch, VR, portable gaming consoles, etc.
**Electronic media use**	These questions cover excessive use of electronic media
**School time**	To indicate the student’s feelings; e.g.; happiness, sadness, etc. at school
**Health and wellbeing**	These questions cover health questions regarding their physical and psychological health
**Sleep Disturbance Scale for ** **Children (SDSC)**	To identify sleeping patterns and problematic sleeping behavior
**Sports and free time**	To indicate participants’ hobbies during leisure time.
**Family and friend**	To indicate the relationship level of participant with family and friends
**Alcohol, tobacco usage**	To assess drinking and smoking patterns
**Noise annoyance and sensitivity**	To indicate noise (from different sources) annoyance at school and home. Also the role of noise in daily life activities disruption
***Home***	
**Electronic media use**	These questions cover engagement in online harassment of others or receiving such messages themselves
**Patient Health Questionnaire-9** ** (PHQ-9)**	To assess the severity of depressive symptoms
**The Generalized Anxiety Disorder** ** (GAD-7)**	To assess anxiety
**Eating Disorder Examination** ** Questionnaire-7 (EDE-Q7)**	to assess body dissatisfaction
**Non-Suicidal Self Injury (NSSI)**	To assess non-suicidal self injury
**Puberty**	To assess the pubertal development
**Health-Related Quality of Life** ** Questionnaire (KIDSCREEN-10** ** Index)**	To assess the general health-related quality of life (HRQoL)
**Selected Zerssen Items**	To assess non-specific somatic symptoms
**The Strengths and Difficulties ** **Questionnaire (SDQ)**	To assess behavioral problems
**Rosenberg Self Esteem**	To assess the self esteem
**Headache Impact Test-6 (HIT-6)**	To assess headache
**School support questions**	To assess the students feeling towards school and if they receive support from students and teachers.
***Interim***	
**Electronic media use**	To assess which devices the adolescents use, mobile phone usage and which platforms adolescents use and how much time they spent
**Reading behaviour**	To assess if adolescents read in their free time
**SDSC**	To assess sleep disturbance and if they use devices before going to bed
**KIDSCREEN-10 Index**	To assess the general health-related quality of life (HRQoL)
** GAD-7**	To assess anxiety

After the school visit, the study participants receive a link via their preferred method of communication, indicated on the consent form. The second part of the questionnaire mainly contains questions on mental health, which the participants may prefer to fill out in their private sphere (e.g., at home) ( Table 4).

In the second school visit, two Noise Sentry RT type-II sound level meters are placed inside each classroom (indoor measurements) and on the façade of the classroom (outdoor measurements) for seven consecutive days to measure environmental noise.

We distribute also interim questionnaire every four months between baseline and follow-up.

The final follow-up examination takes place at school one year after the baseline data collection in the same manner. To ensure low dropout rates, students who have switched classes, location, or are unwell at the date of follow-up are contacted directly using the contact information collected at baseline and suitable arrangements will be made.

***Nested measurement study***

Of the 900 participants enrolled in the main cohort study, a random subsample of 150 students willing to participate are selected for the nested measurement study. Participants indicate in the consent form whether they are interested to participate in the measurement study. A maximum of ten students per school are included in this study. In the event of more than ten adolescents willing to participate in the nested study, a random sample among all volunteers will be selected.

During an instruction visit at school (second visit), the participants are provided with ExpoM-RF4, GENEActiv accelerometer, diary app, and sleepMedia log.

Each student carries an ExpoM-RF4 device for 72 consecutive hours, which should be kept near themselves during school and at home as well as close to the bed when going to sleep. They also record the duration of time they spent in different locations using diary app. After 72 hours, the students have to hand over both devices to the next student. At the end of data collection for two students, the research assistant collects the ExpoM-RF4 and smartphone.

All participants wear a pre-set GENEActiv device on the non-dominant wrist for one week, over 24 hours. They are asked to remove this device during contact sports, where the risk of injuries through the wristband would be too high. In addition to the accelerometer, the study participants fill in a SleepMedia log every morning and evening. After one week, research assistants collect the device and SleepMedia log at the school.

### Online data protection

For data security, all online questionnaires have been built on the Open Data Kit (ODK) platform. This approach allows for end-to-end encrypted survey responses that are directly sent to the secure ODK server at the Swiss Tropical and Public Health Institute (Swiss TPH), thereby ensuring that participant data is protected. Participants undergoing cognitive testing on school computers are provided with a link to the Creyos test battery, which stores results anonymously on an online platform. As Creyos has access to the platform, participants are assigned to a different identification number (ID) and only the study investigators will know which scores belong to which participant (i.e., Creyos will not have access to personal information on any of the participants). Data codes by assigning each participant a unique ID in line with General Data Protection Regulation (GDPR).

### Expected biases

Potential biases in this study are selection bias (due to voluntary participation and later follow up attrition), information bias (related to how accurately and truthfully participants respond to questions in relation to their health and exposure status), residual confounding, and exposure misclassification (also for objectively measured exposures).

We aim to reduce the risk of each bias in numerous ways: by recruiting in many schools and classrooms, i.e.; across educational levels, we increase the diversity of the participants’ backgrounds and therefore sample a roughly representative group of German/English speaking Swiss adolescent students. Follow-up in the same classes after one year (i.e. 8 ^th^ and 9 ^th^ grade) ensures a low attrition rate as seen in the previous two HERMES cohorts, where lost to follow-up was only 6% ( Foerster *et al.*, 2018). We used standardized and validated questionnaires to collect self-reported exposure. Additionally, this self-reported exposure data is complemented with objective measures of classroom noise levels and mobile phone usage provided by mobile phone carrier providers to minimize noise and RF-EMF exposure misclassification. Further, exposure assessment methods are validated with personal RF-EMF measurements in the nested measurement study.

By using a longitudinal design, we aim to overcome many issues associated with cross-sectional design and reverse causality (temporality between exposure-and outcome).

We selected a priori potential confounding factors on which data is being collected in the baseline questionnaire to minimize residual confounding.

### Statistical analysis

***Power and Sample Size***

The sample size of this cohort was determined by a power analysis based on the experience and data distribution of the previous HERMES cohort studies (n=843) on sleep problems, non-specific symptoms, SDQ and cognitive test scores ( Table 5) ( Foerster *et al.*, 2018; Foerster *et al.*, 2019; Roser *et al.*, 2016b). These are conservative calculations, as greater statistical precision can de facto be achieved with continuous exposure-response regression models considering co-variables. Note that in previous analyses various statistical associations were found for emotional and behavioral problems ( Roser *et al.*, 2016a), sleep quality ( Roser *et al.*, 2015b) and cognitive functions ( Foerster *et al.*, 2018) suggesting adequate power with a sample of 900 adolescents.

**Table 5.** Minimal detectable effect per minute increase in daily duration of mobile phone call.

Outcome	Mean score at baseline	standard deviation of change between baseline and follow-up	Minimal detectable change per minute increase of daily mobile phone call duration
**Behavior problems**	9.9	4	0.03
**Sleep**	4.0	2.5	0.02
**Headache**	48	7.4	0.05
**Normalized cognitive score**	0	1	0.007

***Management of personal measurement data***

All collected data of ExpoM-RF4, GENEActiv accelerometer, and app diary data are saved on the Swiss TPH server anonymously and based on a predefined code system. Additionally, SleepMedia log data is transferred from paper-form to an ODK electronic form, based on the unique ID numbers.

**GENEActiv**

Sleep data is extracted from GENEActiv files and integrated with the SleepMedia log data. The GGIR R package is applied to extract sleep data from raw GENEActiv files ( Migueles *et al.*, 2019).

The GGIR package has been developed for GENEActiv accelerometers and uses raw acceleration ENMONZ (Euclidian norm minus one with negative values set to zero) values with validated cut-points to determine the intensity of physical activity ( Hildebrand *et al.*, 2017). The package enables detection of sleep periods by identifying times of sustained inactivity where there is a smaller change in arm angle than a predefined threshold (i.e., a five-degree change in arm angle over a five minute period) ( Van Hees *et al.*, 2015). Sleep detection with these thresholds has been reported to be accurate even without SleepMedia log data ( van Hees *et al.*, 2018).

SleepMedia log data used as an input for the GGIR analysis to guide the accelerometer-based sleep detections.

**ExpoM-RF4 data**

Raw export data of the ExpoM-RF4 needs to be processed and a quality check needs to be performed before final analysis. The following steps are conducted:

Diary correction: At first, the diary app data is merged with the ExpoM-RF4 data based on the time intervals of activities. The plausibility of the diary is first checked by logical rules (e.g. if a participant did not report any travel activity between “home” and “school” or if they logged spending the night at school). Secondly, the consistency between diary data and the location data (GPS, Global Positioning System), collected by the ExpoM-RF4, is checked by a study assistant. In case of inconsistent or incomplete entries, most plausible corrections are applied according to pre-documented method ( Birks *et al.*, 2018; Eeftens *et al.*, 2018a).

Charging correction: during personal measurements, the device needs to be charged daily. Since the ExpoM-RF4 charging cable acts as an FM antenna, the sensitivity to the FM radio and Digital Audio Broadcasting (DAB) bands is erroneously increased. The device logger records the charging process. The exposure data recorded when charging is corrected by substituting the median value during the same activity at the same location while the device was not charging.

Cross-talk correction: A cross-talk error occurs when a signal from one frequency band is unintentionally registered in/as another frequency band, called victim band. This is detected as a temporary correlation between the signals, and corrected by substituting the values of the victim band by the median value during the same activity, but while no cross-talk was registered ( Eeftens *et al.*, 2018b; Schmutz *et al.*, 2022). This is done for the digital enhanced cordless telecommunications (DECT), 1800 MHz downlink, 700 MHz uplink and TDD frequencies.

Band summation: for data analysis, all measured frequency bands are band grouped into broadcast, uplink, downlink (RF-EMF exposure from mobile phone base stations), Wi-Fi, Time division duplex (TDD), DECT as well as total sum of bands ( Table 1).

***Data analyses***

**RF-EMF exposure analysis**

Mean study: For epidemiological analysis among cohort population, cumulative RF-EMF brain dose is the main exposure metric. In addition to cumulative RF-EMF brain dose, modelled far-field RF-EMF exposure from fixed site transmitter is calculated for each participant as time weighted 24h average exposure (mW/m ^2^) using NISmap model for the five activity categories listed above.

Nested measurement study: Personal measurements are descriptively analyzed and illustrated by personal characteristics and by type of activities. For the analysis by personal characteristics, we calculate time-weighted averages to account for differences in measurement periods. To do so, we compute 24 separate hourly averages based on data gathered in each hour, ranging from 01:00 to 02:00, 02:00 to 03:00, and so on. The 24-hour weighted averages are calculated exclusively for individuals who have data for a minimum of 12 separate hours of the day (from 06:00 to 22:00) and at least 6 hours of data at night (from 22:00 to 06:00). These weighted averages are determined by taking the arithmetic mean of the means specific to each of these 24 time slots. For the activity analysis, we determine exposure levels for five activity categories (at home, at school, outdoor, traveling, miscellaneous) reported by the adolescents using the diary app. This is achieved by calculating the average exposure for each individual while engaging in a specific activity. To examine the variations in exposure over different periods, we also compute the average exposure for daytime (06:00–22:00) and nighttime (22:00–06:00), as well as separately for weekdays (Monday to Friday) and weekends (Saturday and Sunday) for each participant.

Mean values of personal measurements calculated per location (e.g. participants bedroom) are also compared with the modelled far-field RF-EMF exposure from fixed site transmitter using correlation coefficients and Kappa coefficients. Factors affecting the agreement between personal measurements and modelling are evaluated by regression modelling.

**eMedia**

For the analysis related to the psychological pathway, various eMedia usage proxies are considered in relation to the RF-EMF emissions involved, such as self-reported and operator reported wireless phone call and data usage (e.g. network technology, number of mobile phone calls, data traffic, social network use, screen time). For every self-reported eMedia exposure variable, we are considered baseline data as well as average duration between baseline and follow-up.

The daily cumulative operator-recorded variables are calculated by summing up all recorded call durations between baseline and follow-up and mean daily usage is computed dividing this sum by the recorded days between baseline and follow-up.

**Noise analysis**

In terms of noise, each transportation noise source i.e.; road, train, and aircraft is treated independently. Different noise exposure metrics are extracted and linked at each residence (home exposure) or school location (school exposure), including the equivalent sound level (L _day_, L _evening_, L _night_), Intermittency Ratio (IR), and Number of Events (N _evt_) ( Wunderli *et al.*, 2016).

We analyzed associations separately for exposures at home and school and a time-weighted total exposure analysis, integrating both home and school exposures.

**Health effects analysis**

The outcome data on cognitive, behavioral, sleep and mental health, as well as non-specific symptoms in association with RF-EMF exposure, eMedia usage and noise is analyzed following an exploratory approach used in the previous HERMES cohort ( Roser *et al.*, 2018; Tangermann *et al.*, 2022; Tangermann *et al.*, 2023) and follows three main analysis approaches:

- *Cross-sectional analysis:* To assess the relationship between exposure and outcomes at baseline. The exposure measures are operator recorded and self-reported eMedia data, modelled far-field RF-EMF exposure from fixed site transmitter, RF-EMF brain dose values and noise. We use mixed models for combined cross-sectional analysis of baseline and follow-up data.
- *Longitudinal analysis:* To understand whether cumulative exposure is followed by a change in outcome. For this analysis, changes in outcomes (difference between follow-up and baseline) are related to baseline exposure (cohort analysis) or change of the RF-EMF exposure measures or eMedia usage, or noise variables between baseline and follow-up investigation (change analysis).
- *Nested cross-sectional analysis:* A cross-sectional analysis of the follow-up outcomes with respect to three days average RF-EMF exposure in the subsample with personal measurements.

The above analyses take into account the multilevel nature of the data (e.g. clustering by school) using mixed linear regression models or generalized linear mixed models, depending on the type of outcome. All models are adjusted for relevant confounders including age, sex, nationality, school level, environmental exposures, frequency of physical activity, alcohol consumption and educational level of the parents. Selection of confounders for various outcome-exposure associations are determined by directed acyclic graphs ( Tennant *et al.*, 2021). Multiple imputations are conducted for missing data.

**Biophysical and the psychological pathways interaction**

Structural equation modelling, as a casual model, is used to differentiate between biophysical and psychological pathways. Given the exploratory approach, we refrain from hypothesis testing but interpret results in terms of consistency for various outcomes and exposure measures.

**International level analysis**

HERMES3 cohort in Switzerland along with INMA (INfancia y Medio Ambiente) Project from Spain, NINFEA (Nascita e INFanzia: gli Effetti dell’Ambiente) from Italy, REPRO_PL (Polish Mother and Child Cohort Study) from Poland, ABCD study (The Amsterdam Born Children and their Development), from the Netherlands as well as two studies from Japan and South Korea follow the same protocol and questionnaires to collect exposures and outcomes data in baseline and one year later. All data from different cohorts will be pooled together to increase the statistical power. Cohorts from Spain, Poland and the Netherlands already have some data on eMedia use, outcomes, and covariates of interest, collected at earlier ages. Within the GOLIAT project, NINFEA and HERMES3 cohorts performed two additional follow-up assessments, specifically investigating eMedia use and the selected outcomes.

### Dissemination of the study findings

The findings from this study will be published as preprints and then disseminated through peer reviewed publications and conference presentations. The results of the study may also be shared with relevant mental health organizations and used to inform future research. The findings may be shared with stakeholders and politicians who set the RF-EMF regulations for Switzerland.

## Preliminary results

At the time of submission (04.15.2024) we have contacted 277 schools and of them, 24 are participating, 10 are maybe participating, 137 do not have an answer yet and 106 do not want to participate

Totally, 181 adolescents have agreed to participate in the main study and 53% of them have agreed to be included in the nested measurement study. Sleep and RF-EMF assessment of 70 participants have been completed so far and recruitment for the main and measurement study is still ongoing. A sample data on the RF-EMF personal measurement and GENEActiv sleep assesment have been presented to reflect a general view of the objective data.

**Figure 3.**
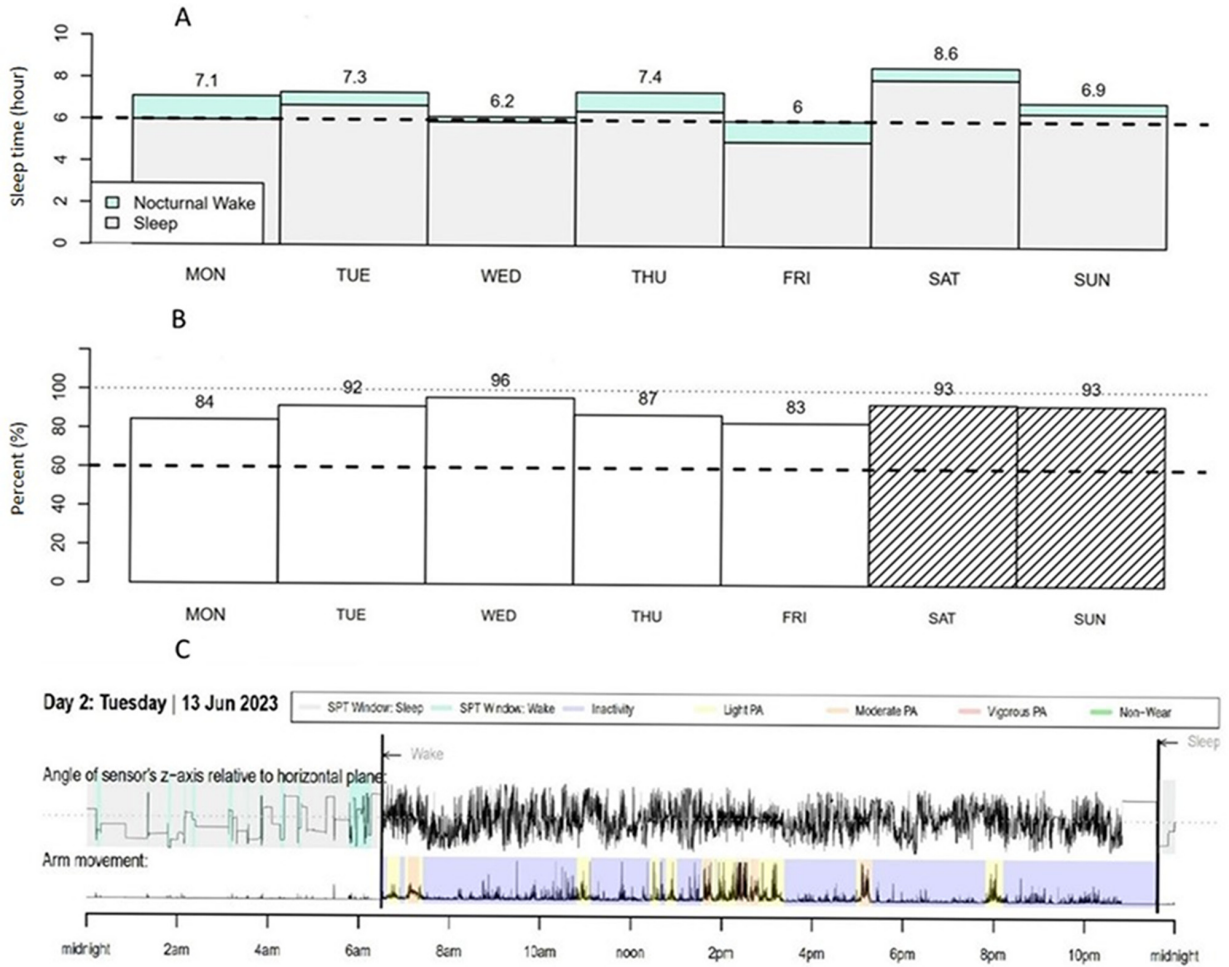
Example of GENEActiv data of one participant: ( **A**) Sleep duration and ( **B**) sleep efficiency for a week, and ( **C**) one day sleep highlight and physical activity. *SPT: sleep tracked, PA: physical activity.

Figure 3 illustrates the first analysis of sleep of a participant in the measurement study. Average sleep duration was 7.1 h and average sleep efficiency was 90% per night.

**Figure 4.**
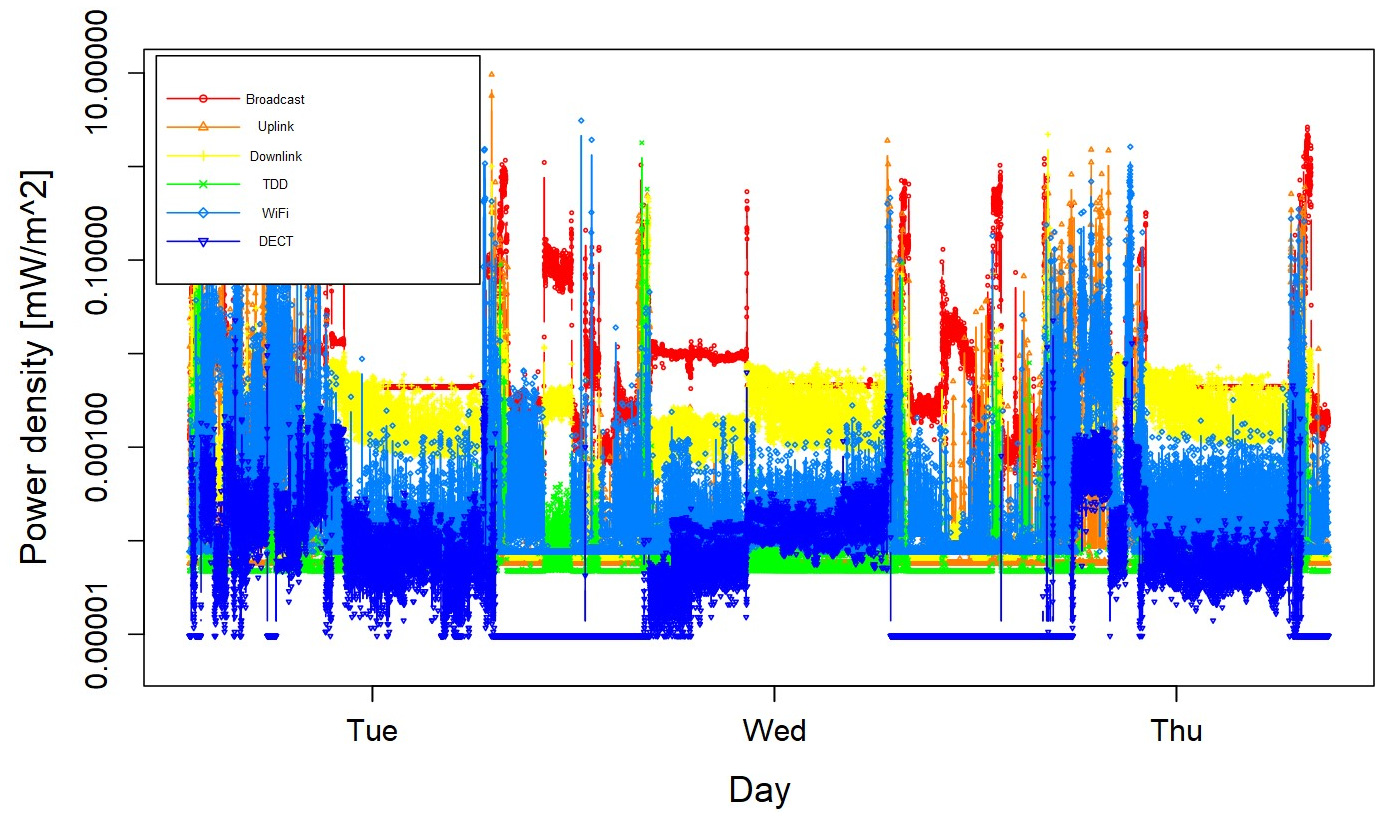
Radiofrequency electromagnetic field exposure profile of a participant in measurement study.

Figure 4 shows the RF-EMF exposure profile of one participant. The mean and median of total exposure to RF-EMF were 0.04 and 0.007 mW/m ^2^.

## Conclusion

The HERMES3 cohort is a continuation of research on eMedia use transportation noise and adolescents’ health in Switzerland ( Roser *et al.*, 2015b; Roser *et al.*, 2016a; Roser *et al.*, 2016b; Schoeni *et al.*, 2015), reflecting the current state of eMedia and mobile phone use as well as noise exposure in adolescents and their association with cognitive, behavior, sleep and mental health, as well as non-specific symptoms.

Our study is strengthened by its prospective design and the use of objective exposure and outcome data ascertained from mobile phone providers, EMF and noise exposure modeling and measurements as well as actigraphy and cognitive test battery, all of which have a minimal burden on the participants but significantly strengthen our findings.

Overall, this project provides, together with other cohorts of the GOLIAT project, significant inputs and scientific value in RF-EMF field and potential future impact on regulation/policies. Today, mobile phones are among the most frequently used devices for eMedia usage, with over 97% of Swiss adolescents owning a smart phone and spending a daily average of over three hours on weekdays and over five hours on weekends on these devices for activities like browsing the internet, video gaming, and using SNS ( Bernath *et al.*, 2020). Given the widespread usage of eMedia, disentangling pathways on how mobile phone and eMedia use may affect health of adolescents is vital. Fears around RF-EMF exposure and controversies surrounding 5G are of significant public and political concern, and our study provides more information in this field allowing policymakers and the public to make better-informed decisions. There is also significant societal value in better understanding the effects of eMedia usage on mental health issues among adolescents, as these constitute approximately 13% of the burden of disease in 10–19 year olds ( WHO, 2021) and over 50% of mental health disorders have their age of onset before 15 years ( Kim-Cohen *et al.*, 2003). Indeed, a recent nationally-representative survey found that among Swiss adolescents, nearly half reported low emotional wellbeing, one third were classified as depressed, and one fourth classified as living with moderate to severe anxiety ( Barrense-Dias *et al.*, 2021). It is important to deepen our understanding of the potential risks associated with mobile phone and eMedia usage, especially considering the swift expansion of the eMedia landscape in recent years.

## Ethics and consent

All study procedures are non-invasive and pose minimal risk to study participants. The HERMES3 cohort is undertaken in accordance with the seventh revision (2013) of the Helsinki Declaration on medical research involving human subjects and the study protocol has been approved by the ethics committee Ethikkommission Nordwest- und Zentralschweiz (EKNZ) under registration number “BASEC 2022-02185”. Both adolescents and their primary guardians should provide their written consent in order to be eligible to participate in the study.

## Data Availability

This paper is a study protocol, therefore no data are associated with it. Access to the cohort data is restricted to the core research team. External requests for access to anonymized data will be considered after completion of the study and subsequent primary publications. The requests must comply with data protection regulations and the objectives and methods of the proposed research must be scientifically and ethically sound.
