# Supplementary material for: Prospective cohort study on non-specific symptoms, cognitive, behavioral, sleep and mental health in relation to electronic media use and transportation noise among adolescents (HERMES): study protocol
Source: Open Res Eur. 2025 Nov 24;4:120. Originally published 2024 Jun 19. [Version 2] doi: 10.12688/openreseurope.17667.2 (PMC13098621; doi:10.12688/openreseurope.17667.2)
Supplement: Peer review history

# Reviewer report: Reviewer response for version 2

**DOI:** 10.21956/openreseurope.23796.r64759

**Published:** 2025-12-27

**Authors:** Payam Sajadi

**Affiliations:** 1 Geography, Maynooth University Department of Geography (Ringgold ID: 442382), Maynooth, County Kildare, Ireland

No further comments to make

Is the study design appropriate for the research question?

Yes

Is the rationale for, and objectives of, the study clearly described?

Yes

Are sufficient details of the methods provided to allow replication by others?

Partly

Are the datasets clearly presented in a useable and accessible format?

Yes

Reviewer Expertise:

Geospatial analyis, Machine Learning, Remote Sensing

I confirm that I have read this submission and believe that I have an appropriate level of expertise to confirm that it is of an acceptable scientific standard.

# Reviewer report: Reviewer response for version 2

**DOI:** 10.21956/openreseurope.23796.r64758

**Published:** 2025-12-16

**Authors:** Atanu Kumar Pati

**Affiliations:** 1 Pt. Ravishankar Shukla University, Raipur, India

I found out that the revisions are appropriate. Therefore, I recommend indexing of the protocol article.

Is the study design appropriate for the research question?

Partly

Is the rationale for, and objectives of, the study clearly described?

Yes

Are sufficient details of the methods provided to allow replication by others?

Partly

Are the datasets clearly presented in a useable and accessible format?

Not applicable

Reviewer Expertise:

NA

I confirm that I have read this submission and believe that I have an appropriate level of expertise to confirm that it is of an acceptable scientific standard.

# Reviewer report: Reviewer response for version 1

**DOI:** 10.21956/openreseurope.19092.r59841

**Published:** 2025-10-06

**Authors:** Payam Sajadi

**Affiliations:** 1 Geography, Maynooth University Department of Geography (Ringgold ID: 442382), Maynooth, County Kildare, Ireland

Reviewer Report

 Overall, this is a well-written and important study protocol addressing the potential health impacts of electronic media use, RF-EMF exposure, and transportation noise among adolescents. The study is ambitious and timely, given the increasing relevance of these exposures and their implications for public health. The design, with repeated follow-ups and objective measurement subsamples, is a major strength. However, there are several areas where the manuscript could be improved for clarity, transparency, and completeness. My comments below are intended to be constructive and to help strengthen the paper further.

 1. Exposure Assessment:

 The protocol nicely integrates both RF-EMF, emedia and transportation noise exposures. However, for RF-EMF, it remains somewhat unclear how well the modelling approach will capture individual-level variability. Could the authors clarify how these limitations will be addressed, and whether calibration/validation against the personal measurement subsample is planned?

 2. Temporal Resolution:

 Given the rapid changes in adolescent media use, the 4-month intervals between follow-ups are appropriate. Still, recall bias may affect self-reported eMedia use. Did the authors consider incorporating objective logging data (e.g. from smartphones) where feasible?

 3. Confounding & Effect Modification:

 Given the multifactorial nature of the studied outcomes, confounding is a major concern. Could the authors clarify how they plan to control for these potential confounders?

 4. Longitudinal Retention:

 Adolescent cohorts often face high attrition. Could the authors discuss strategies to maintain engagement and minimize loss to follow-up?

 5. Clarity of Aims:

 The study aims are ambitious and span multiple exposures and outcomes. It might strengthen the protocol to specify primary vs. secondary outcomes, to avoid dilution of statistical power and interpretability.

 6. Recent Literature & Update of Protocol:

 The protocol is clear and detailed, but I think the paper needs to be updated based on the most recent status of the study.

 7. Pathway Analysis:

 The protocol mentions interest in “biophysical and psychological pathways.” It would be useful if the authors could outline in more detail how these pathways will be operationalized in the analyses.

 8. Noise Exposure Modelling:

 Transportation noise is modelled based on home and school addresses. However, adolescents may spend substantial time in other environments. Could the authors comment on possible exposure misclassification and how this will be addressed?

 9. Ethics & Data Privacy:

 The study involves adolescents, repeated follow-up, and potentially sensitive health data. Could the authors provide more detail on ethical approvals, data privacy safeguards, and how informed consent from both parents and adolescents is managed?

 10. Generalizability:

 The cohort is based in Switzerland, which may have specific cultural and technological contexts. Could the authors comment on how far the findings may be generalized to other countries or contexts?

Is the study design appropriate for the research question?

Yes

Is the rationale for, and objectives of, the study clearly described?

Yes

Are sufficient details of the methods provided to allow replication by others?

Partly

Are the datasets clearly presented in a useable and accessible format?

Yes

Reviewer Expertise:

Geospatial analyis, Machine Learning, Remote Sensing

I confirm that I have read this submission and believe that I have an appropriate level of expertise to confirm that it is of an acceptable scientific standard, however I have significant reservations, as outlined above.

# Response

**Published:** 2025-11-20

**Authors:** Hamed Jalilian

**Affiliations:** Epidemiology and Public Health, Swiss Tropical and Public Health Institute, Basel, Allschwil, Switzerland

**General comment:** Overall, this is a well-written and important study protocol addressing the potential health impacts of electronic media use, RF-EMF exposure, and transportation noise among adolescents. The study is ambitious and timely, given the increasing relevance of these exposures and their implications for public health. The design, with repeated follow-ups and objective measurement subsamples, is a major strength. However, there are several areas where the manuscript could be improved for clarity, transparency, and completeness. My comments below are intended to be constructive and to help strengthen the paper further. **Response: ** We sincerely thank the reviewer for the positive and encouraging feedback. We appreciate the recognition of the study’s relevance, design, and methodological strengths. We also thank the reviewer for the constructive suggestions provided, which have been carefully considered to further improve the clarity, transparency, and completeness of the manuscript.  

**Comment 1:** 1. Exposure Assessment: The protocol nicely integrates both RF-EMF, emedia and transportation noise exposures. However, for RF-EMF, it remains somewhat unclear how well the modelling approach will capture individual-level variability. Could the authors clarify how these limitations will be addressed, and whether calibration/validation against the personal measurement subsample is planned?

**Response 1: ** Far-field RF-EMF exposure models cannot capture all individual variability. To address this, we will compare modelled exposure with data from the personal exposimeter subsample, allowing us to quantify and adjust for potential biases. We added this information to “Exposure assessment” heading, *“RF-EMF exposure”* section. Note that personal measurements are not necessarily the gold standard as they are affected by near field sources and body shielding.  

**Comment 2:** 2. Temporal Resolution: Given the rapid changes in adolescent media use, the 4-month intervals between follow-ups are appropriate. Still, recall bias may affect self-reported eMedia use. Did the authors consider incorporating objective logging data (e.g. from smartphones) where feasible?

**Response 2: ** We agree that recall bias may influence self-reported electronic media use. To address this limitation, we will attempt to validate self-reported eMedia use using operator-recorded data, which include information on network technology, data traffic, and call duration obtained from mobile phone operators. While direct smartphone logging was not feasible due to privacy and resource constraints, combining questionnaire data with operator-based usage records will help improve the accuracy and reliability of exposure assessment. We added this information to “Exposure assessment” heading, *“eMedia usage”* section.  

**Comment 3:** 3. Confounding & Effect Modification: Given the multifactorial nature of the studied outcomes, confounding is a major concern. Could the authors clarify how they plan to control for these potential confounders?

**Response 3: ** We fully acknowledge the multifactorial nature of the studied outcomes and the need to control for potential confounding and effect modification. As described in the Statistical Analysis and Covariates sections, we plan to adjust for a wide range of relevant variables, including sociodemographic factors (age, sex, socioeconomic status, parental education), lifestyle factors (physical activity, caffeine use), and environmental factors (noise exposure, urbanization level, and school characteristics) (see Table 3). We will assess potential effect modification by variables such as sex and socioeconomic status using interaction terms. Additionally, multi-level mixed-effects models will be applied to account for clustering within schools. This comprehensive approach aims to minimize confounding and provide robust effect estimates for the associations of interest.  

**Comment 4:** 4. Longitudinal Retention: Adolescent cohorts often face high attrition. Could the authors discuss strategies to maintain engagement and minimize loss to follow-up?

**Response 4: ** We acknowledge that maintaining participant engagement in adolescent cohorts can be challenging. But actually, by doing the school setting we are not faced with this challenge. A total of 242 participants took part in and completed the school-based follow-up (response rate 83%). To minimize attrition, we implement several retention strategies, including:

- Incentives such as 5 CHF gift and personalized feedback summaries to encourage continued participation.
- Consistent involvement of school staff to support coordination and promote participation.
- Flexible scheduling of follow-up assessments to accommodate school timetables and students’ availability.

We added these items to the “Recruitment, screening and informed consent procedure” heading, last paragraph.  

**Comment 5:** 5. Clarity of Aims: The study aims are ambitious and span multiple exposures and outcomes. It might strengthen the protocol to specify primary vs. secondary outcomes, to avoid dilution of statistical power and interpretability.  

**Response 5: ** Given the lack of a priori biological hypotheses for the outcomes we consider the study to be explorative in nature. Self-reported and measured sleep quality cognitive tests and mental health indicators are primary outcomes while behavior and non-specific symptoms are secondary outcomes.  

**Comment 6:** 6. Recent Literature & Update of Protocol: The protocol is clear and detailed, but I think the paper needs to be updated based on the most recent status of the study.

**Response 6: ** The manuscript has been updated to reflect the most recent status of the study, including recruitment progress, data collection updates, and minor methodological clarifications, while keeping the original protocol framework unchanged.  

**Comment 7:** 7. Pathway Analysis: The protocol mentions interest in “biophysical and psychological pathways.” It would be useful if the authors could outline in more detail how these pathways will be operationalized in the analyses.

**Response 7: ** We have expanded the description of the biophysical and psychological pathways in the revised manuscript. The biophysical pathway now focuses on physiological effects of cumulative RF-EMF exposure and noise, while the psychological pathway addresses behavioral and emotional aspects of eMedia use (e.g., nighttime use, type of social networking). We have also specified that these pathways will be analyzed using structural equation modeling (SEM) and mediation analysis to identify direct and indirect effects on health outcomes. We added the new update to “statistical analysis” heading, *“Biophysical and the psychological pathways interaction”* section.  

**Comment 8:** 8. Noise Exposure Modelling: Transportation noise is modelled based on home and school addresses. However, adolescents may spend substantial time in other environments. Could the authors comment on possible exposure misclassification and how this will be addressed?

**Response 8: ** It is true that adolescents spend time outside their home and school, such as during commuting, sports, or social activities, which could lead to some exposure misclassification. However, home and school addresses are likely to capture the majority of daily noise exposure, as these are the locations where adolescents spend most of their time and are most relevant in terms of experienced distress from noise. Other locations tend to be more sporadic or shorter in duration, so their contribution to average long-term exposure is expected to be relatively small. It is standard in noise research to only consider noise at home and at work.  

**Comment 9:** 9. Ethics & Data Privacy: The study involves adolescents, repeated follow-up, and potentially sensitive health data. Could the authors provide more detail on ethical approvals, data privacy safeguards, and how informed consent from both parents and adolescents is managed?

**Response 9: ** To improve clarity and transparency, we revised and reorganized the relevant sections in the manuscript. We have updated “Online data protection” heading with providing more detailed description of the data handling process, including secure storage procedures, coding and pseudonymization of participant IDs, and data protection measures for each data source (questionnaires, cognitive testing, actigraphy, RF-EMF, and operator data). It also clarifies the roles of external partners and the use of encrypted data transfers compliant with Swiss and EU (GDPR) regulations. The “Ethics” section has been aligned in tone, keeping information on informed consent procedures, parental consent, participant rights, and ethics committee approval. These revisions make the manuscript more concise, transparent, and consistent with journal formatting requirements while fully addressing data privacy and ethical safeguards.  

**Comment 10:** 10. Generalizability: The cohort is based in Switzerland, which may have specific cultural and technological contexts. Could the authors comment on how far the findings may be generalized to other countries or contexts?

**Response 10: ** We acknowledge that the study is conducted within the Swiss context, which may differ from other countries in terms of cultural habits, school systems, and technological infrastructure. Nevertheless, the exposures of interest, electronic media use, RF-EMF exposure, and transportation noise, are globally relevant, and the methodological framework used in this study is applicable in other settings. While absolute exposure levels and behavioral patterns may vary internationally, the underlying mechanisms and analytical approach can be generalized and replicated in other populations. We have added this statement to the end of “ *Discussion*” section.

# Reviewer report: Reviewer response for version 1

**DOI:** 10.21956/openreseurope.19092.r46574

**Published:** 2024-12-06

**Authors:** Atanu Kumar Pati

**Affiliations:** 1 Pt. Ravishankar Shukla University, Raipur, India

In the article, titled “Prospective cohort study on non-specific symptoms, cognitive, behavioral, sleep, and mental health in relation to electronic media use and transportation noise among adolescents (HERMES): study protocol,” the authors propose a rigorous study protocol to evaluate the effects of electronic media use and transportation noise among non-specific symptoms, cognitive function, behavior, sleep, and mental health in a population of adolescents. There are several positive points in the protocol. The protocol simultaneously addresses diverse outcomes in the realms of cognition, behavior, sleep, and mental health of adolescents. 

 However, several issues diminish the approach. I have outlined those issues below for the authors to take note of.

 1.    The sample size is precariously small and may not be adequate for generalization. There were only 24 schools, out of 277 contacted, that participated in this study by the time this article was submitted (15th April 2024).

 2.    Further, the sample size was only 70 when this article was submitted. It implies that the participation of the adolescents was only about 39% of the projected sample. This is indeed too little for validation of any protocol.

 3.    I didn’t understand why French-speaking adolescents were not included in the study.

 4.    The authors had input from only 24 schools till the submission of this article. But authors did not mention anything about the school start time of all those schools. It is well known that school start time has a prominent effect on the sleep quality and quantity of school students. Earlier school start times can lead to chronic sleep loss and disrupt circadian rhythms. Further, later start times can lead to better behavioral health and fewer risk-taking behaviors, such as bullying and fighting. Therefore, it is important to consider school start time as an important factor.

 5.    Why cognitive tests were not conducted 4- and 8 months after the initiation of the study? 

 6.    Did the authors conclude that sleep duration has declined in the studied participants? 

 7.    At what time of the day the cognitive variables were measured? As I understand the cognitive tests were conducted during the school visit by the investigators. It would be appropriate to mention if tests were conducted at the beginning, middle, or towards the end of school time. I mentioned this to emphasize that a circadian rhythm in various types of cognitive functions has been demonstrated in humans.

 8.    Did the authors try to find out if any gadgets emitting RF-EMF were installed in the bedrooms of the participants?

 9.    How many participants’ houses were very close to a BTS?

 10.    In the conclusion section, less emphasis has been given to the impacts of transportation noise. Are the schools located very close to the railway networks and airport/flight corridors? It is not unusual to think that there may be a distinct variability among the schools concerning the distance of schools from railway networks and flight corridors. 

 11.    I am surprised that the authors have chosen to publish their findings before completing their pilot study. While the study protocol is well-prepared, supporting it with such limited data raises some concerns.

Is the study design appropriate for the research question?

Partly

Is the rationale for, and objectives of, the study clearly described?

Yes

Are sufficient details of the methods provided to allow replication by others?

Partly

Are the datasets clearly presented in a useable and accessible format?

Not applicable

Reviewer Expertise:

Chronobiology, Animal Behavior and Physiology

I confirm that I have read this submission and believe that I have an appropriate level of expertise to confirm that it is of an acceptable scientific standard, however I have significant reservations, as outlined above.

# Response

**Published:** 2025-11-20

**Authors:** Hamed Jalilian

**Affiliations:** Epidemiology and Public Health, Swiss Tropical and Public Health Institute, Basel, Allschwil, Switzerland

**General comment: ** In the article, titled “Prospective cohort study on non-specific symptoms, cognitive, behavioral, sleep, and mental health in relation to electronic media use and transportation noise among adolescents (HERMES): study protocol,” the authors propose a rigorous study protocol to evaluate the effects of electronic media use and transportation noise among non-specific symptoms, cognitive function, behavior, sleep, and mental health in a population of adolescents. There are several positive points in the protocol. The protocol simultaneously addresses diverse outcomes in the realms of cognition, behavior, sleep, and mental health of adolescents. 

**Response:** We thank the reviewer for the positive feedback and appreciation of our comprehensive approach addressing multiple adolescent health outcomes.  

**Comment 1:** 1.    The sample size is precariously small and may not be adequate for generalization. There were only 24 schools, out of 277 contacted, that participated in this study by the time this article was submitted (15th April 2024).

**Response1:** The number of participating schools (24 out of 277 contacted) reflected the recruitment status at the time of manuscript submission, while data collection is still ongoing. Since then, recruitment has progressed, with 27 schools participating in the study and approximately 2,000 students have been invited. Among them, 305 students and their guardians have provided consent to participate in the main study. We agree that the sample size is on the small size. As written in the “statistical analysis” heading, “ *international level analysis” *section, the data will be pooled with other cohorts from GOLIAT to increase the statistical power. The main purpose of this protocol paper is to describe the study design and methodology rather than to generalize findings. We have updated the number of participants in the “Preliminary results” section.  

**Comment 2:** 2.    Further, the sample size was only 70 when this article was submitted. It implies that the participation of the adolescents was only about 39% of the projected sample. This is indeed too little for validation of any protocol.

**Response 2:** The number of 70 participants reflected an early stage of data collection at the time of submission. Recruitment has continued thereafter, and the study has now reached 305 adolescents with parental consent and 292 completed baseline questionnaires, with follow-up assessments ongoing. As this article presents the study protocol, its purpose is to outline the methodological framework rather than to validate results based on preliminary data.  

**Comment 3:** 3.    I didn’t understand why French-speaking adolescents were not included in the study.

**Response 3:** Due to limited resources, we are unable to provide a French-speaking investigator, which restricts recruitment to English/German-speaking adolescents. Expanding the study to French-speaking regions would require additional staff and logistical support beyond the current project scope.  

**Comment 4:** 4.    The authors had input from only 24 schools till the submission of this article. But authors did not mention anything about the school start time of all those schools. It is well known that school start time has a prominent effect on the sleep quality and quantity of school students. Earlier school start times can lead to chronic sleep loss and disrupt circadian rhythms. Further, later start times can lead to better behavioral health and fewer risk-taking behaviors, such as bullying and fighting. Therefore, it is important to consider school start time as an important factor.

**Response 4:** Although we did not collect the official school start times, schools in our study generally begin classes around 08:00 a.m. and finish by 04:00 p.m., which is consistent across participating schools. We have added this information to “Study procedure” heading, *“Main study”* section. In the main cohort, we assess relevant aspects of sleep timing and quality through questionnaire items during weekdays and weekends, difficulty waking up in the morning, and feeling tired upon waking. In the measurement subsample, we also obtain objectively measured wake-up times over one week using sleep monitoring device. Therefore, while the precise school start time is not directly recorded, its potential impact on sleep duration and circadian rhythm is indirectly captured through these self-reported and objectively measured sleep indicators. Moreover, differences between weekday and weekend sleep variables will allow us to examine social jet lag, which may reflect circadian disruption associated with school schedules.  

**Comment 5:** 5.    Why cognitive tests were not conducted 4- and 8 months after the initiation of the study? 

**Response 5: ** There are three main reasons why cognitive tests are conducted only at baseline and at the one-year follow-up. First, due to limited resources and logistical constraints, it is not feasible to repeat the full set of cognitive assessments at 4- and 8-month intervals. Second, several schools are unable or unwilling to accommodate additional testing sessions within their academic schedules. Third, we were most interested in medium to long-term associations and not in acute associations after a few weeks. Therefore, cognitive testing is restricted to baseline and the one-year follow-up to ensure high data quality and compliance.  

**Comment 6:** 6.    Did the authors conclude that sleep duration has declined in the studied participants? 

**Response 6: ** As the manuscript is a study protocol, no data analyses or conclusions have yet been conducted. The paper’s purpose is to describe the study design, objectives, and planned methods, including how sleep duration and quality will be assessed, rather than to report findings. Analyses of changes in sleep duration will be presented in future publications once data collection and processing are complete.  

**Comment 7:** 7.    At what time of the day the cognitive variables were measured? As I understand the cognitive tests were conducted during the school visit by the investigators. It would be appropriate to mention if tests were conducted at the beginning, middle, or towards the end of school time. I mentioned this to emphasize that a circadian rhythm in various types of cognitive functions has been demonstrated in humans.

**Response 7: ** The cognitive tests are conducted during regular school hours, typically between 8:00 a.m. and 4:00 p.m., mentioned in the revised version, depending on each school’s schedule and class availability Although the exact timing varies across schools, testing takes place under comparable daytime conditions within this time window. We have added this clarification to the revised manuscript and will consider the timing as an additional variable in the analysis  

**Comment 8:** 8.    Did the authors try to find out if any gadgets emitting RF-EMF were installed in the bedrooms of the participants?

**Response 8: ** We did not specifically and directly assess the installation of RF-EMF emitting-gadgets in participants’ bedrooms, but we do collect related information through questionnaire items. For example, participants are asked: *“Do you have a device in the bedroom while you are sleeping (e.g., mobile phone, laptop, tablet)?*” and “ *Do you use another mobile device (e.g., virtual glasses, portable gaming consoles, augmented reality devices)*?” These questions help capture personal use and presence of electronic devices that may contribute to RF-EMF exposure before sleep.  

**Comment 9:** 9.    How many participants’ houses were very close to a BTS?

**Response 9: ** We do not examine the participants’ neighborhood characteristics. RF-EMF exposure is measured objectively using the ExpoM-RF4 personal exposimeter, which records exposure levels across multiple mobile communication frequency bands. However, we did not specifically collect questionnaire data on the proximity of base transceiver stations (BTS) to participants’ homes. In addition, RF-EMF exposure modeling is conducted based on the BTS antenna locations surrounding each participant’s home, allowing us to estimate environmental exposure levels more comprehensively. Note it is well known that distance to base station is not a good predictor for exposure.  

**Comment 10:** 10.    In the conclusion section, less emphasis has been given to the impacts of transportation noise. Are the schools located very close to the railway networks and airport/flight corridors? It is not unusual to think that there may be a distinct variability among the schools concerning the distance of schools from railway networks and flight corridors. 

**Response 10: ** In our sample the proportion of participants with high exposure to railway or aircraft noise is very small and the most relevant noise source is road traffic noise. Our analyses will further adjust for major confounders (in particular socioeconomic position) and relevant environmental co-exposures to minimize such kind of bias.  

**Comment 11:** 11.    I am surprised that the authors have chosen to publish their findings before completing their pilot study. While the study protocol is well-prepared, supporting it with such limited data raises some concerns.

**Response 11: ** We did not report any preliminary or inferential results in this protocol paper. The few sample results included are presented only as illustrative examples to demonstrate the structure and type of data obtained (e.g., sleep and RF-EMF measurements) and to help readers understand the planned analytical approach. The purpose of this article is to describe the study design and methodology, not to interpret or validate findings.
